# Cardiopulmonary disease as sequelae of long-term COVID-19: Current perspectives and challenges

**DOI:** 10.3389/fmed.2022.1041236

**Published:** 2022-11-30

**Authors:** Rudolf K. F. Oliveira, Peter S. Nyasulu, Adeel Ahmed Iqbal, Muhammad Hamdan Gul, Eloara V. M. Ferreira, John William Leclair, Zin Mar Htun, Luke S. Howard, Ana O. Mocumbi, Andrew J. Bryant, Jacques L. Tamuzi, Sergey Avdeev, Nicola Petrosillo, Ahmed Hassan, Ghazwan Butrous, Vinicio de Jesus Perez

**Affiliations:** ^1^Division of Respiratory Diseases, Department of Medicine, Federal University of São Paulo (UNIFESP), São Paulo, Brazil; ^2^Division of Epidemiology and Biostatistics, Department of Global Health, Faculty of Medicine and Health Sciences, Stellenbosch University, Stellenbosch, South Africa; ^3^National Health System (NHS), Global Clinical Network, London, United Kingdom; ^4^Department of Internal Medicine, University of Kentucky, Lexington, KY, United States; ^5^Department of Medicine, Stanford University, Stanford, CA, United States; ^6^Division of Pulmonary and Critical Care, National Institute of Health, University of Maryland, College Park, College Park, MD, United States; ^7^National Heart and Lung Institute, Imperial College London, London, United Kingdom; ^8^Faculty of Medicine, Universidade Eduardo Mondlane, Maputo, Mozambique; ^9^Non-communicable Diseases Division, Instituto Nacional de Saúde, Marracuene, Mozambique; ^10^College of Medicine, University of Florida, Gainesville, FL, United States; ^11^Department of Pulmonology, I.M. Sechenov First Moscow State Medical University (Sechenov University), Moscow, Russia; ^12^Infection Prevention and Control-Infectious Disease Service, Foundation University Hospital Campus Bio-Medico, Rome, Italy; ^13^Department of Cardiology, Cairo University, Cairo, Egypt; ^14^Medway School of Pharmacy, University of Kent at Canterbury, Canterbury, United Kingdom; ^15^Division of Pulmonary, Allergy and Critical Care Medicine, Stanford University Medical Center, Stanford, CA, United States

**Keywords:** COVID-19, pulmonary hypertension, interstitial lung disease, thrombosis, right ventricular dysfunction

## Abstract

COVID-19 infection primarily targets the lungs, which in severe cases progresses to cytokine storm, acute respiratory distress syndrome, multiorgan dysfunction, and shock. Survivors are now presenting evidence of cardiopulmonary sequelae such as persistent right ventricular dysfunction, chronic thrombosis, lung fibrosis, and pulmonary hypertension. This review will summarize the current knowledge on long-term cardiopulmonary sequelae of COVID-19 and provide a framework for approaching the diagnosis and management of these entities. We will also identify research priorities to address areas of uncertainty and improve the quality of care provided to these patients.

## Introduction

The post-COVID-19 syndrome (or long COVID) is defined by the persistence of symptoms with a history of probable or confirmed SARS-CoV-2 infection, usually 3 months from the onset and lasts at least 2 months in the absence of an alternative diagnosis (1). CDC defines long COVID in the context of lingering symptoms that can last greater than 4 weeks and even months in a patient who had the onset of COVID-19 infection 4 weeks prior (2). The main symptoms are fatigue, muscle weakness, dyspnea, sleep, and cognitive disturbances. The proposed hypotheses to explain these findings relate to the viral toxicity itself, systemic inflammatory response, persistent impairment of gas exchange, restrictive lung disease, perfusion abnormality due to micro and macro vascular thrombosis, chronic myocardial dysfunction, corticosteroids use, prolonged hospitalization with immobility, and post-traumatic stress syndrome (3, 4).

Even though the burden of long COVID symptoms correlates with the severity of COVID-19 acute infection (5, 6), patients with milder COVID-19 also have a substantial burden of long COVID symptoms on follow-up. In a study that compared COVID-19 patients who were hospitalized vs. home-isolated patients at 6 months, the persistence of symptoms was noted in 81 vs. 55% of patients respectively (7). In a clinical follow-up of 150 non-critically ill COVID-19 patients after 3 months, 66% of the patients reported symptoms—40% reported asthenia, 30% reported dyspnea, and 23% reported anosmia or dysgeusia (8). In a multistate telephonic survey of 274 patients who had been tested positive for COVID-19 as outpatient, 35% of the patients had not returned to their usual state of health when interviewed 2–3 weeks after being tested positive (9).

A significant economic burden on the healthcare system is expected with the increasing long COVID cases, and further studies exploring this aspect are encouraged. The full spectrum and burden of long COVID in terms of symptoms and cardiopulmonary sequelae such as pulmonary hypertension will be more apparent in the coming years. This narrative review will focus on the most prevalent cardiopulmonary sequelae reported in COVID-19 survivors. We will address the current understanding of the pathobiological mechanisms potentially involved in developing these conditions and focus on areas of uncertainties that should be prioritized in research efforts. We will also review the management strategies for these commonly encountered post-COVID-19 conditions. As the pool of patients recovering from COVID-19 continues to increase, healthcare providers will need to learn to recognize these cardiopulmonary sequelae early and develop a management plan, including focusing on rehabilitation techniques that will prevent further deterioration and improve the quality of life for these patients.

## Long-term cardiovascular complications in COVID-19

Cardiovascular complications in COVID-19 infection, including myocardial injury, myocarditis, heart failure, and arrhythmias, have been well-reported during the acute phase since the pandemic’s start (10). The incidence of long-term cardiovascular complications after 30 days of COVID-19 was studied in the US database on a cohort of 153,760 veterans, and an increased risk of strokes, dysrhythmias, myocarditis, ischemic heart diseases, heart failure, cardiac arrest, pulmonary embolism, and deep vein thrombosis was observed (11). The higher risk of cardiovascular complications also extended to COVID-19 survivors who were not hospitalized in this large study (11). These findings were comparable to the results from another large retrospective cohort study (12). In another study of 587 COVID-19 patients, who were followed 1 year after discharge, 11 patients were thought to have died from complications due to COVID-19, including cardiovascular complications such as acute myocardial infarction, acute heart failure and sudden death related to malignant arrhythmia and pulmonary thromboembolism (13). Additionally, new-onset diabetes and major cardiovascular adverse events have also been reported in COVID-19 patients after hospital discharge (14). Regarding the dynamic changes in the ECG or arrhythmias observed during the acute phase of COVID-19, they tend to resolve by 6 months post-COVID-19 (15, 16). However, sinus tachycardia is prevalent in post-COVID-19 survivors (15, 17) and postural orthostatic tachycardia may be the etiology in the setting of persistent complaints of dizziness (18). The cardiovascular impact of COVID-19 infection has also been studied in autopsy studies.

### Risk of myocarditis in post-COVID-19 survivors

In an extensive multicenter study screening for myocarditis in 1,597 athletes who underwent cardiac magnetic resonance (CMR) imaging, 2.3% were diagnosed with COVID-19 myocarditis—9 had clinical and 27 had subclinical myocarditis. Follow-up CMR in the 4–14 weeks demonstrated resolution of T2 elevation in all 27 athletes and late gadolinium enhancement in 11 athletes (3). In another observational cohort study following athletes, clinically indicated CMR had a higher yield of myocarditis [15 of 119 (12.6%)] vs. primary screening CMR [6 of 198 (3.0%)]. In post-COVID-19 patients with cardiac symptoms, cardiac edema (54%) and late gadolinium enhancement (31%) on CMR imaging has been reported (15). However, in a study involving 32 patients post-COVID-19 with persistent cardiovascular symptoms, only 3 (9%) met the criteria of acute myocarditis on CMR imaging, and none of those patients met criteria for myocarditis on endomyocardial biopsy (19). In a meta-analysis, the overall prevalence of myocarditis in athletes who had recovered from COVID-19 ranged from 1 to 4% (20). In a study of post-COVID-19 patients (average time since COVID-19 was 4 months) where myocarditis was diagnosed with biopsy in six patients, SARS-CoV-2 was found in four biopsies, lymphocytic myocarditis was found in five, and one patient had giant cell myocarditis (21). The presence of COVID-19 in myocardial tissue months after infection with COVID-19 explains the persistence of chronic inflammation in patients with myocarditis. In a meta-analysis of 277 post-mortem examinations, COVID-19 related histopathological changes such as macro or microvascular thrombi, inflammation, or intraluminal megakaryocytes were common; however, the true prevalence of myocarditis was likely less than 2% based on the pathological analysis. Global uniformity with the use of an autopsy checklist was suggested in reporting cardiovascular pathology findings in COVID-19 (22).

These data indicate that post-COVID-19 survivors can have lingering cardiovascular morbidities in the long-term. Increased burden of cardiovascular complications is also found in COVID-19 patients who may not be hospitalized for COVID-19. During the clinic’s follow-up visits of COVID-19 patients, the possibility of long-term cardiovascular complications should be entertained, especially in the presence of symptoms, and referred to the appropriate specialty clinic when needed. Long-term cardiovascular complications with increased risk in COVID-19 are presented in Table 1.

**TABLE 1:** Long-term cardiovascular complications with increased risk in COVID-19.

Arrhythmias; atrial fibrillation, sinus tachycardia, sinus bradycardia, atrial flutter, ventricular arrhythmias
Inflammatory heart diseases; myocarditis, pericarditis
Ischemic heart disease: acute coronary syndrome, myocardial infarction, ischemic cardiomyopathy, angina
Other cardiac disorders: heart failure, non-ischemic cardiomyopathy, cardiac arrest
Thrombotic complications; DVT, pulmonary embolism, superficial venous thrombosis
Cerebrovascular disorders; stroke, TIA

## Right ventricular dysfunction

### Prevalence and pathogenesis of right ventricular dysfunction

Right ventricular dysfunction is a known cardiovascular complication of COVID-19 infection observed in 20–31% of cases and associated with increased mortality (23–27). Additionally, worse right ventricular function is associated with elevated troponin and worse clinical presentation (23) and impaired longitudinal strain on echocardiography (27, 28). In a meta-analysis of 1,450 patients, those with right ventricular dysfunction had twofold mortality compared to those without (48.5 vs. 24.7%) (29).

**FIGURE 1:**
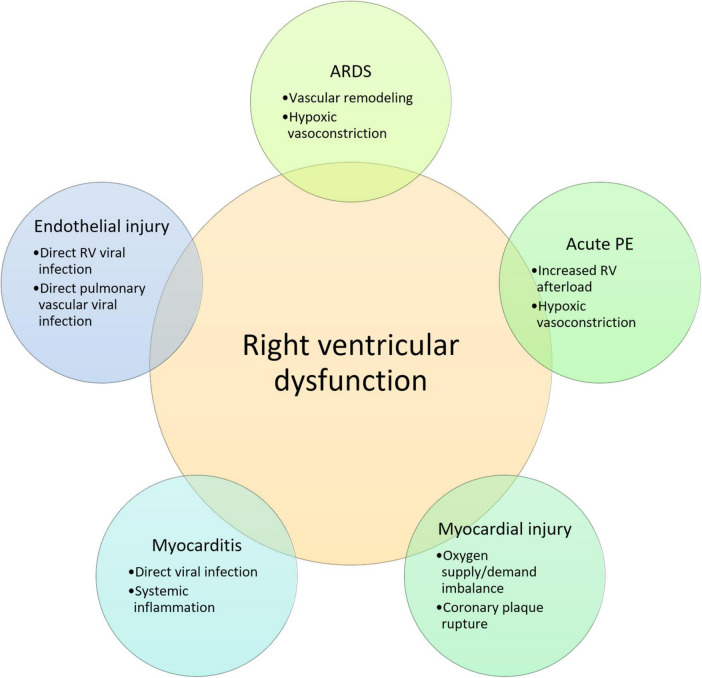
Potential mechanisms of right ventricular dysfunction in COVID-19. RV, right ventricular; ARDS, acute respiratory distress syndrome; PE, pulmonary embolism; IL, interleukin.

It is known that the thin-walled right ventricle is susceptible to ischemia and dysfunction in response to sudden increases in afterload or coronary occlusion, which in turn may compromise left ventricular function (30). Increased right ventricular afterload in SARS-CoV-2 infection can be secondary to pulmonary parenchymal abnormalities combined with macro- or micro pulmonary vascular disease. In this context, the extent of local tissue damage and the cytokine storm triggered by the host immune response may contribute to the severity of the disease and right ventricular dysfunction (31). Furthermore, activation of the inflammatory cells present in atherosclerotic plaques may lead to coronary plaque rupture and subsequent myocardial ischemia (32). Finally, cardiac CMR imaging findings of patients recovered from COVID-19 infection point to myocardial tissue abnormalities and impaired right ventricular function in otherwise healthy subjects, ultimately suggesting chronic cardiac disease as a consequence of SARS-CoV-2 infection (33). Potential mechanisms of right ventricular dysfunction in COVID-19 are summarized in Figure 1.

### Management of right ventricular dysfunction

Management of acute right ventricular dysfunction depends on treating the underlying etiology in addition to the optimization of the intravascular volume, reducing the right ventricular afterload, and enhancing ventricular contractility. Milrinone, a phosphodiesterase-3 inhibitor, enhances right ventricular contractility and reduces pulmonary vascular resistance (34) and may be used instead of dobutamine; however, it might induce hypotension and arrhythmias (35). Inhaled nitric oxide, a selective pulmonary vasodilator, may be beneficial in reducing and stabilizing the pulmonary artery systolic pressure and might reduce the risk of right ventricular failure in COVID-19 (36). Levosimendan, a calcium sensitizer with the advantages of improving the right ventricular contractility and reducing right ventricular afterload with no increase in myocardial oxygen consumption (37), may increase cardiac output and decrease mean pulmonary artery pressure, right atrial pressure, and peripheral systemic resistance in the setting of acute heart failure. Nevertheless, no randomized controlled trials support improved mortality (38–41). Finally, in the presence of severe right ventricular dysfunction, venoarterial, venovenous, venovenous-arterial, or venopulmonary-arterial extracorporeal membrane oxygenation may be required to augment the right ventricle function (42, 43).

In the setting of chronic right heart failure, long-term supplemental oxygen use is recommended for patients with resting or exercise-induced hypoxemia, aiming to reduce hypoxic vasoconstriction and avoid an increase in pulmonary vascular resistance (44). Volume overload should be treated with diuretics. In the setting of isolated right heart failure due to pulmonary embolism, while being cautious of avoiding volume depletion, low-dose diuretics may be used (45). In the setting of decreased left ventricular ejection fraction, goal-directed medical therapy should be employed (46). Whether pulmonary vasodilators can be used or not depends on the primary etiology of pulmonary hypertension, which is further discussed in the section of post-COVID-19 pulmonary hypertension.

Right ventricular dysfunction may not be fully reversible in some COVID-19 survivors, depending on the chronicity of the ischemic insult. In a canine study that involved banding of the pulmonary artery inducing right heart failure, reversibility of the right ventricle was shown to be more profound in those in which pulmonary afterload was reduced early (47). In another canine study, depressed right ventricular function due to severe pulmonary artery obstruction was restored after right coronary artery hyper perfusion (48). However, chronic hypoxia in rats has been shown to induce non-reversible right ventricular dysfunction (49). Additionally, direct or indirect injury of right ventricular myocytes and endothelial cells by SARS-CoV-2 or systemic inflammation might chronically impact right ventricular myocardial and endothelial viability resulting in right ventricular dysfunction. Also, we should point out that the development of chronic thromboembolic pulmonary hypertension (CTEPH) can be accompanied by right ventricular dysfunction (50), which is further discussed in the section of post-COVID-19 pulmonary hypertension. Further studies on the reversibility of right ventricular dysfunction in COVID-19 patients should be pursued and patients with right ventricular dysfunction will require close follow-up for signs of heart failure.

## Post-COVID-19 lung fibrosis

### Overview of lung function recovery post-COVID-19

A subgroup of post-COVID-19 patients will evolve with persistent chest imaging abnormalities (51) and/or reduced lung diffusion capacity (52, 53). Additionally, post-COVID-19 patients reporting persistent dyspnea, are more likely to have a restrictive pattern, lower carbon monoxide diffusion capacity, reduced functional capacity, and increased exertional desaturation (54). There findings were confirmed by a systematic review of studies assessing pulmonary function post-COVID-19, where altered carbon monoxide diffusion capacity, restrictive pattern, and obstructive pattern were observed in 39, 15, and 7% of patients, respectively, between 1 and 3 months after COVID-19 (55).

In a large study undertaken during the first wave of the COVID-19 epidemic in Wuhan, China, 1,733 of 2,469 discharged patients with COVID-19 were followed up 6 months after hospital discharge. Patients with higher severity scores were found to have lower 6-min walking distance, decreased carbon monoxide diffusion capacity on the pulmonary function test, and worse imaging findings corroborated by high computed tomography (CT) scores (56). Although the pathological characterization of postmortem lung samples from patients who died early after COVID-19 has been extensively reported (57, 58), little is known about the residual pathological changes in the lungs of patients within 2 years of survival of acute COVID-19 (11).

Taken together, these studies suggest that patients with greater severity of acute COVID-19 may have a higher risk for long-term pulmonary complications. These persisting abnormalities are attributed to diffusion impairment and structural pulmonary abnormalities such as pulmonary fibrosis (59).

### Evolving pulmonary fibrotic changes in COVID-19

Pulmonary fibrosis is the consequence of aberrant wound healing, which results in a cascade of pathological changes that replaces the lung parenchyma with an extracellular matrix (60). On imaging, pulmonary fibrosis is suggested by the parenchymal bands, reticular opacities, traction bronchiectasis, and honeycombing (61). Pulmonary fibrosis is among the most feared chronic pulmonary complications of COVID-19. It can be challenging to separate the fibrotic changes from the reversible lingering opacities from COVID-19 pneumonia based on the imaging in the post-COVID-19 setting. Often encountered are the imaging findings denoting “grey areas,” which represent evolving immature fibroblastic changes in the background of the diffuse alveolar damage (DAD) remodeling over time (62). It is thus preferred to use the term post-COVID interstitial lung disease (PCILD), which covers a broader spectrum of evolving pulmonary changes seen in patients who have recovered from COVID-19 pneumonia and leaves the prospect of reversibility an open question (62, 63).

Reticular changes suggestive of fibrotic changes were found in half of COVID-19 survivors (23 out of 46) screened at 2 weeks intervals after the onset of severe COVID-19, and these changes persisted at the 4-week follow-up (64). Han et al. showed fibrotic changes in 35% of survivors of severe COVID-19 pneumonia at a 6-month follow-up chest CT (61). According to Vasarmidi et al., the rate of COVID-induced fibrosis may exceed 30% (65, 66). Wu et al. demonstrated that 24% of patients had abnormal CT images at 12 months (67). Similarly, Huang et al. found that at 2 months post-discharge, extensive fibrosis was evident on the CT imaging of 42 out of 81 (52%) patients (68).

The prevalence of PCILD disease varied across all these studies. While interpreting the results of these studies, it is essential to realize that in post-SARS-CoV-2 respiratory syndrome, CT findings suggestive of fibrosis at initial imaging may eventually improve or even resolve with further follow-up (69, 70), but several cases of progressive pulmonary fibrosis have been described in patients with COVID-19 (71–73). Based on the observational studies on SARS-CoV-1, the residual lung damage decreased by the end of the first year (74, 75); however, it persisted after that in the 15 years of follow-up (76). Given that post-COVID-19 pulmonary fibrosis can result in severe chronic hypoxic respiratory failure with significantly debilitating dyspnea, patients with irreversible PCILD after approximately a year may be potential candidates for a lung transplant. Flaifel et al. described the lung pathology of such a population of patients before the lung transplant (77). At 8–11 months after COVID-19 diagnosis, the significant changes noted were described as proliferative and fibrotic phases of DAD, diffuse type 2 pneumocyte hyperplasia, prominent interstitial capillary neo-angiogenesis, and mononuclear cells, specifically macrophages (77).

### Risk factors of pulmonary fibrosis in COVID-19

Numerous risk factors have been attributed to the development of PCILD, such as the length of stay in the hospital and the ICU, the use of high-flow nasal oxygen, mechanical ventilation, and the occurrence of acute respiratory distress (66, 78). Acute respiratory distress syndrome (ARDS) is a condition well known for high rates of development of pulmonary fibrosis (79–81). Ventilator-associated lung injury, in the setting of non-adherence to protective lung strategies, can further worsen lung injury (82). Among survivors of severe COVID-19, 20% of non-mechanically ventilated and 72% of mechanically ventilated individuals had fibrotic-like radiographic abnormalities 4 months after hospitalization (83). Greater initial severity of the disease and a longer duration of mechanical ventilation were independent risk factors for the development of fibrosis-like abnormalities. Similar findings were also reported in another study that found most COVID-19 patients with pulmonary fibrosis (81%) during acute COVID-19 infection were admitted to an ICU, and 63% required mechanical ventilation (84).

Patient-related risk factors included male gender, older age, active smoking, persistent breathlessness, and alcohol abuse (68, 85–88). Men are three times more likely to develop PCILD (85). Han et al. identified age >50 years and heart rate >100 beats per minute at admission as independent predictors of fibrotic-like changes in survivors of severe COVID-19 pneumonia at a 6-month follow-up (61). Finally, cytokines such as interleukin-6 and upregulation of other growth factors such as TGF-β1, FGF, and EGF also contribute to the development of pulmonary fibrosis (89, 90). Neutrophil extracellular traps play a key role in the interplay between inflammation and thrombotic changes in the lung. They may have a role in the development of lung fibrosis (91) and therefore can be a potential therapeutic target (92).

### Link between idiopathic pulmonary fibrosis and COVID-19

Gene-environment interactions and genetic susceptibility factors may play a role in the development of PCILD. Fadista et al. found a genetic correlation between idiopathic pulmonary fibrosis and COVID-19 severity, pointing several variants associated with both increased idiopathic pulmonary fibrosis risk and increased risk of severe COVID-19 (93). Additionally, genome-wide association studies have identified multiple genetic signals associated with severe COVID-19, including a variant within the DPP9 gene related to increased idiopathic pulmonary fibrosis risk (94). Four genetic association signals showed evidence of a shared causal variant between idiopathic pulmonary fibrosis and at least one COVID-19 phenotype, namely loci at 7q22.1, near MUC5B, near ATP11A, and near DPP9 (94). Finally, shorter blood leukocyte telomere lengths are independent risk factors for developing fibrotic-like abnormalities in COVID-19 (83). Thus, this genomic biomarker may predict increased susceptibility to the development of post-COVID-19 pulmonary fibrosis.

### Diagnosis of post-COVID interstitial lung disease

A diagnosis of PCILD should be based on clinical, radiologic, and pathologic findings. While the appropriate timing for the diagnosis of irreversible fibrosis has not yet been established, serial evaluation with lung function test, including assessment for carbon monoxide diffusion capacity and 6-min walk test, can be tailored to the patient’s clinical course, symptoms, and oxygen requirement. Further evaluation may involve the need for a chest CT (90, 95). Earlier in the pandemic, more frequent serial evaluations at 3, 6, 9, and 12 months were recommended, as there was a need for further research studies in this area (95, 96). Although there is no specific finding from laboratory testing, immunohistochemical analysis of TGF-b, IL-1α, and IFN-β may play a role in predicting PCILD. In this context, a recent study found that high IL-1α and TGF-β and low plasma levels of IFN-β could predict an increased relative risk of lung fibrosis-like changes in PCILD (97). More research is needed to confirm the prognostic role of genetic tests such as telomere shortening in PCILD.

### Management of post-COVID interstitial lung disease

The role of treatment with antifibrotic and anti-inflammatory drugs (65, 98–100) in improving PCILD symptoms remains unclear and inconclusive. There is still little data on the safety and effectiveness of those treatments in COVID-19 patients with PCILD, and most clinical trials have yet to be completed. Recent evidence has shown that the use of high-dose vs. low-dose prednisolone in a randomized control trial of 130 patients with PCILD did not show significant improvement in symptoms after 6 weeks of follow-up (101). In contrast, three observational studies have reported improvement with glucocorticoids in symptomatic patients with PCILD (102–104). However, these findings should be interpreted cautiously because the two studies had small sample sizes (103, 104). A recent trial found that Pycnogenol^®^ and Centellicum^®^ may improve the residual clinical picture in PCILD patients and reduce the number of subjects progressing to lung fibrosis (100). However, this result should be viewed in the context of small sample size and a poorly designed study. Randomized clinical trials with Pycnogenol^®^ and Centellicum^®^ in PCILD patients are highly recommended. Other suggested therapies include mesenchymal stem cells, cytokine Inhibitors, spironolactone, TGF-β1 Inhibitors, CD147 Inhibitors, poly-(ADP-Ribose) polymerase Inhibitor, galectin-3 (e.g., BIO 300), and Chinese medicine drugs for pulmonary fibrosis in convalescent sequelae of COVID-19 (99, 105).

Presently, the treatment for PCILD remains supportive. Referral to pulmonary rehabilitation programs and evaluation for supplemental oxygen therapy should be considered for patients meeting the criteria. The role of treatment with antifibrotic drugs remains unclear whose lung function continues to deteriorate.

## Persisting coagulation abnormalities in COVID-19 survivors

Various coagulation abnormalities such as increased D-dimer, fibrinogen, factor VIII levels, mild thrombocytopenia, and slightly prolonged prothrombin time have been noted in the setting of the inflammatory milieu featured in COVID-19 (106–110). These coagulation abnormalities predispose COVID-19 patients to acute macro and micro thromboembolic events (111). The predisposition to coagulopathy is manifested in the form of microangiopathy with widespread thrombosis observed in COVID-19 autopsied lungs (112). The 90-day incidence rate of venous thromboembolism may range from 0.2 to 0.8% in COVID-19 cases and up to 4.5% in hospitalized patients (113).

Persistent hypercoagulability has been observed in COVID-19 survivors. A study on 208 COVID-19 survivors 2 months after onset, identified significant activation of endothelial cells and *in vivo* thrombin generation in at least one out of four COVID-19 survivors (114). At 3 months, 203 COVID-19 survivors were found to have increased endothelin-1, thrombin-antithrombin complex, von-Willebrand factor, and inflammatory cytokines (115). Others have identified increased thrombin generation capacity and hypofibrinolytic activity in the setting of increased Factor VIII levels and decreased plasminogen-activator inhibitor 1 (116). In 39 COVID-19 survivors followed for coagulation abnormalities after a year, elevated D-dimer, factor VIII, von-Willebrand factor antigen and interleukin-6 was reported. In a prospective registry study of 4,906 hospitalized patients followed at the mean of 92 days, venous thromboembolic event rates of 1.55% were noted, more than half of which included pulmonary embolism (117). Other smaller studies had thromboembolic events ranging from 0.2 to 2.5% (118–121). Thus, persisting hypercoagulopathy and higher rates of pulmonary thromboembolism have been noted in the post-discharge period in COVID-19 survivors.

## Pulmonary hypertension

The vascular remodeling and luminal microthrombi noted in acute SARS-CoV-2 infection raise the suspicion that SARS-CoV-2 infection could be a risk factor for the future development of pulmonary arterial hypertension (122). Thickened pulmonary vascular walls, one of the hallmarks of pulmonary arterial hypertension, were reported in SARS-CoV-2 infection. In an autopsy study, the pulmonary vascular wall thickness was more than twice thicker than those of patients who died from H1N1 influenza (123).

### The role of ACE2 in the pathogenesis of pulmonary vascular disease in COVID-19

The vascular endothelium is one of the primary targets of SARS-CoV-2 and the molecular pathways and cellular abnormalities observed in SARS-CoV-2 pulmonary vascular injury are similar to the pathogenesis pathway of pulmonary arterial hypertension (124). Direct viral infection and inflammatory cytokines outbursts are plausible mechanisms for endothelial damage caused by SARS-CoV-2. The host receptor for the virus (ACE2) is widely expressed in endothelial cells. Monteil et al. showed that SARS-CoV-2 can directly infect engineered human blood vessels, which can be inhibited by human recombinant soluble ACE2 (125). Varga et al. demonstrated in an autopsy study that viral particles were present in the endothelial cells of the glomerular capillary loops by electron microscopy of kidney tissue (126).

The presence of ACE2 within normal levels in the lung seems to be essential to combat inflammatory lung disease (127). In PAH, angiotensin II is upregulated and its level correlates with disease severity. Downregulation of ACE2 during SARS-CoV-2 infection can potentially increase angiotensin II circulating levels. Perivascular lymphocytic infiltration has been found in lung biopsies from patients with SARS-CoV-2 infection (125, 126). An autopsy study by Ackermann et al. demonstrated severe endothelial injury in SARS-CoV-2 infection, mediated by the entry of the virus into the endothelial cells, resulting in micro and macrovascular thrombosis. These features are distinct compared to patients who died from ARDS secondary to H1N1 influenza (112). The mechanisms described earlier may also contribute to developing pulmonary arterial hypertension in COVID-19 survivors.

### Pulmonary fibrosis and development of pulmonary hypertension in COVID-19

**FIGURE 2:**
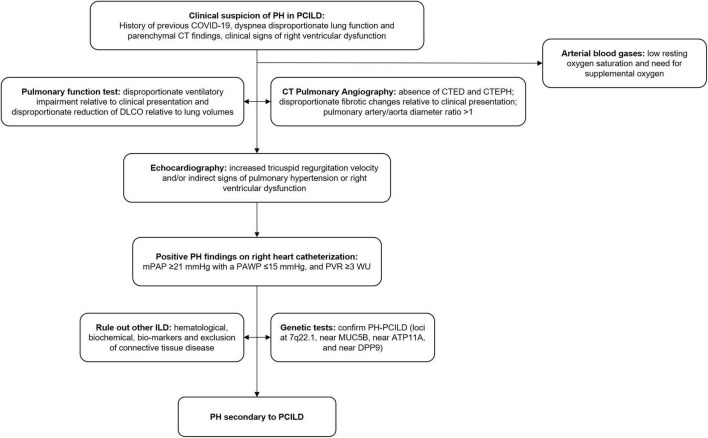
Proposed algorithm for the diagnosis of pulmonary hypertension in PCILD. PH, pulmonary hypertension; PCILD, post-COVID-19 interstitial lung disease; CT, computed tomography; DLCO, diffusion capacity for carbon monoxide; CTED, chronic thromboembolic disease; CTEPH, chronic thromboembolic pulmonary hypertension; mPAP, mean pulmonary arterial pressure; PAWP, pulmonary arterial wedge pressure; PVR, pulmonary vascular resistance; ILD, interstitial lung disease.

Pulmonary fibrosis can be further complicated by pulmonary hypertension (25), which in turn has been noted to be a major determinant of higher mortality (26). The severity of pulmonary fibrosis is not correlated with the development of pulmonary hypertension, and numerous mechanisms involving dysregulation of molecular pathways resulting in vascular remodeling have been suggested (25). Vascular remodeling is now considered an essential contributor to pulmonary hypertension besides the traditionally known factors of hypoxic vasoconstriction and capillary bed destruction in pulmonary fibrosis. Loss of BMPR2 signaling, upregulation of A2BAR, and endothelial-to-mesenchymal transition have been speculated to be involved in the vascular remodeling process in pulmonary fibrosis (23, 27). The entry of the virus into the endothelial cells of pulmonary capillaries has been implicated in the vascular remodeling in COVID-19. The incidence of pulmonary hypertension in PCILD needs to be studied further. In general, transthoracic echocardiography in chronic lung diseases is insufficient to confirm or rule out pulmonary hypertension (29). Right heart catheterization is considered the gold standard for diagnosing pulmonary hypertension in this population (29). A proposed algorithm for the diagnosis of pulmonary hypertension in PCILD is shown in Figure 2.

### Chronic thromboembolic disease and chronic thromboembolic pulmonary hypertension in COVID-19

While most acute pulmonary embolisms and clots resolve with anticoagulation, clot persistence can lead to continued post-embolic symptoms of shortness of breath and the development of chronic thromboembolic disease (CTED). About 30–50% of the patients have persistent defects up to 1 year after diagnosis (128). CTEPH refers to the development of pulmonary hypertension in the setting of CTED. CTEPH is estimated to have a 0.5–5% prevalence after PE (129–132). In the European CTEPH registry, pulmonary endarterectomy mortality rate was 4.7% (133). Additionally, untreated or undiagnosed CTEPH patients have a poor prognosis and a mean pulmonary arterial pressure >30 mmHg is associated with mortality rates >50% in 10 years (134).

In COVID-19 patients, persistent coagulation abnormalities have been noted 4 months after discharge (116, 135), making COVID-19 patients further prone to CTED. Endothelial dysfunction, which can lead to inflammation and thrombosis, is the common pathology in COVID-19 and CTEPH (136). This supports the concept of *in situ* thrombosis in COVID-19, which is a different phenotype than traditional venous thromboembolism (137). The histological evaluation of lungs in COVID-19 patients showed pulmonary vascular endotheliitis with widespread micro thrombosis (112). Whether severe COVID-19 patients without acute pulmonary embolism and ongoing long-term respiratory symptoms may have developed pulmonary hypertension in the setting of micro-thrombosis needs further evaluation. Dyspnea and prolonged hypoxia being common post-COVID-19 symptoms due to other etiologies such as PCILD, make an early diagnosis of COVID-19-related CTED and CTEPH challenging.

In the absence of scientific evidence specific to COVID-19 for CTED and CTEPH, we recommend standard follow-ups for all patients diagnosed with acute pulmonary embolism during the first 2 years. These patients should be monitored for recurrent thromboembolism and right heart failure symptoms and the optimal timing for initiating diagnostic testing is 3–6 months after acute pulmonary embolism diagnosis (138). Lung ventilation/perfusion scintigraphy (V/Q scan) is the screening test of choice (139). However, residual pulmonary consolidations and fibrosis from post-ARDS changes from COVID-19 might cause abnormalities in ventilation and make interpretation challenging. In such patients, CT pulmonary angiography is a reasonable alternative imaging modality (139, 140). Right heart catheterization is the gold standard confirmation test for CTEPH. Where available, referral to CTEPH centers should be initiated simultaneously with workup once the diagnosis is suspected.

**FIGURE 3:**
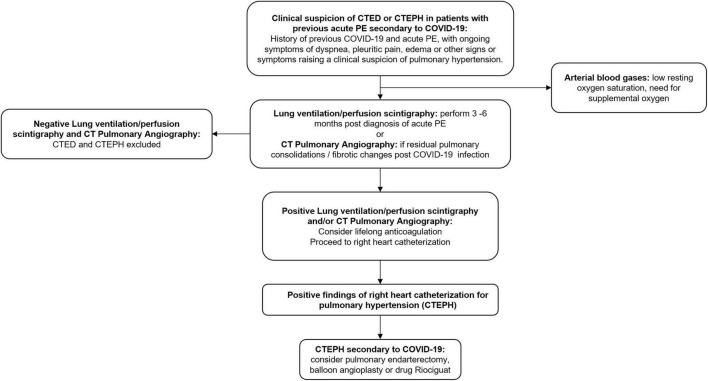
Proposed algorithm for investigation and management of chronic thromboembolic disease and chronic thromboembolic pulmonary hypertension secondary to COVID-19. CTED, chronic thromboembolic disease; CTEPH, chronic thromboembolic pulmonary hypertension; PE, pulmonary embolism; CT, computed tomography.

Presently, there are no evidence-based guidelines on the optimal management of CTED and CTEPH in COVID-19 survivors. Most societies recommend a minimum of 3 months of anticoagulation for acute pulmonary embolism and lifelong anticoagulation for CTED or CTEPH (139, 141, 142). If CTEPH is the clinical diagnosis, then pulmonary endarterectomy is the definitive therapy for CTEPH (139). Percutaneous balloon angioplasty shows benefit for inoperable patients and patients with residual CTEPH after endarterectomy (143, 144). For those patients not eligible for endarterectomy or angioplasty, Riociguat is the drug of choice (145). A proposed algorithm for CTED and CTEPH in post-COVID-19 patients is shown in Figure 3.

### Post-COVID-19 pulmonary hypertension

Although theoretically, it is expected that post-COVID-19 patients can develop new onset pulmonary hypertension in the setting of interstitial lung disease and pulmonary vasculopathy; however, literature on post-COVID-19 pulmonary hypertension remains scant. We speculate that the complete picture of the burden of post-COVID-19 pulmonary hypertension will be more apparent in the upcoming years, as was the case with HIV and the use of anti-obesity drugs. Additionally, the interaction between parasites such as schistosomiasis in endemic areas and COVID-19 in the development of pulmonary hypertension would be worth exploring further.

Post-COVID-19 pulmonary hypertension has only been mentioned in the setting of case-reports so far. Cueto-Robledo et al. described a case of the development of severe pulmonary hypertension in a patient 3 months post-COVID-19. The finding of pulmonary trunk dilatation on imaging and the echocardiographic results prompted right heart catheterization, which confirmed severe pulmonary hypertension (146). In another case, new pulmonary hypertension was diagnosed on right heart catheterization 6 weeks after discharge from COVID-19 (147). In cases of severe pulmonary hypertension, right heart catheterization will be essential to confirm the pressure readings in the pulmonary vasculature and also to determine further if there is a component of WHO group 1 pulmonary arterial hypertension in addition to WHO group 3 pulmonary hypertension due to PCILD. Pulmonary arterial hypertension-specific therapy or pulmonary arterial vasodilators are rarely indicated in group 3 pulmonary hypertension unless the right heart catheterization findings and pulmonary function testing, demonstrate pulmonary hypertension out of proportion to the chronic lung disease (146, 148). Such patients should be referred to pulmonary hypertension centers for expert-opinion.

## The role of pulmonary rehabilitation in post-COVID-19

### Pathogenesis of exercise intolerance in post-COVID-19

Most of the evidence regarding the etiology of exercise intolerance in post-COVID syndrome comes from the cardiopulmonary exercise tests, which have been summarized in Table 2. Based on these studies, it has been suggested that exercise intolerance could result from deconditioning, defined as loss of physical fitness due to the inability to maintain an optimal level of physical activity or training (16, 149–158). In cardiopulmonary exercise findings, deconditioning is the reduction of peak oxygen uptake in the absence of known central and peripheral cardiocirculatory diseases. However, post-COVID-19 exercise intolerance may be attributed to impaired peripheral oxygen utilization or extraction due to mitochondrial injury. Evaluating patients with persistent symptoms after COVID-19 infection, Singh et al. elegantly demonstrated through an invasive cardiopulmonary exercise test that oxygen delivery was normal; however, reduced peripheral oxygen extraction and uptake were noted, indicating lower diffusive oxygen delivery to the mitochondria (159). Corroborating peripheral muscle impairment in the setting of myopathy due to inflammatory changes and mitochondrial cellular respiration dysfunction, a case series of 16 patients post-COVID-19 who were evaluated for fatigue and myalgia and who performed muscle biopsy, had muscle fiber atrophy (38%) with signs of regeneration (56%) and mitochondrial and inflammatory changes(62%) (160). This may be the result of myopathy because of viral injury, which may be responsible for the persistence of fatigue in long COVID. Nonetheless, a more recent report demonstrated that in post-COVID-19 patients with fatigue vs. non-fatigue, the only difference in cardiopulmonary exercise test was lower peak oxygen uptake (ml/kg/min), without other noticeable differences in exercise responses (161).

**TABLE 2:** Mechanisms of exercise intolerance in post-COVID syndrome.

Time of the evaluation after hospitalization-sample (*n*)	Comparison subgroups	Findings
Gao et al. (182) 1-month post-discharge follow-up (*n* = 10)	Pre-rehabilitation patients with post-COVID-19	● Reduction in _PEAK_V′O_2_ 66.2 ± 10.5% pred (*n* = 10) ● Decreasing oxygen pulse relative to predicted values (*n* = 7) ● DLCO <80% (*n* = 3) and high V′E/V′CO_2_ at AT (*n* = 2)
Raman et al. (16) >2–3 months of disease-onset (*n* = 58)	Moderate to severe COVID-19 vs. controls	● 54.9% of patients (_PEAK_V′O_2_ <80% pred) ● Lower _PEAK_V′O_2_ ● Lower OUES ● Higher V′E/V′CO_2_ (worse in MRI lung parenchymal abnormalities, and it correlated with markers of inflammation)
Liu et al. (183) 7 months (*n* = 41)	Persistence or absence of pulmonary fibrosis on chest CT	● _PEAK_V′O_2_: 16.4 ± 3.6 ml/kg/min (with fibrosis) vs. 20.2 ± 3.7 ml/kg/min (no fibrosis) ● Older and more severe hospitalization ● Lower _PEAK_V′O_2_ ● Lower METS ● Higher V′E/V′CO_2_
Debeaumont et al. (153) 6 months (*n* = 23)	ICU vs. ward	● 52% of patients (_PEAK_V′O_2_ <85% prev) ● Higher ΔV′E/ΔV′CO_2_
Dorelli et al. (184) 5 months (*n* = 28)	ΔV′E/ΔV′CO_2_ > 31 or ≤31	● Mean _PEAK_V′O_2_: 29.2 ± 8.3 ml/kg/min ● No difference in pulmonary function variables at rest and in CPET responses
Baratto et al. (162) At time of hospital discharge (*n* = 18)	COVID-19 vs. control participants	● _PEAK_V′O_2_ 59% pred (IQR 32) ● Lower slope V′O_2_/WR 8.1 (1.2) ml/min/W ● Lower O_2_ pulse 9.1 (2.0) beat/L ● Higher V′E/V′CO_2_ 40 (9.0) related to hypocapnia ● Lower Ca-vO_2_/CaO_2_: 0.66 (0.19) ● Higher VD/VT at rest with elevated VD/VT during exercise
Rinaldo et al. (150) 3 months (*n* = 75)	Reduced or normal _PEAK_V′O_2_	● 55% of patients (_PEAK_V′O_2_ <85% prev) ● Lower lactate threshold ● Lower ΔV′O_2_/ΔWR ● Lower pulse O_2_
Skjørten et al. (151) 3 months (*n* = 156)	Post-COVID and normal population without COVID-19 by *z*-score (20% in ICU)	● 31% of patients (_PEAK_V′O_2_ <80% prev) ● 15% reduced lactate threshold ● 16% ventilatory limitation ● 23% desaturation >4% ● 15% increased ΔV′E/ΔV′CO_2_
Motiejunaite et. (152) 3 months (*n* = 114)	DLCO ≤ or >75% prev	● 75% of patients (PEAKV′O2 <85% prev) ● Lower PEAKV′O2 ● Lower lactate threshold ● Tendency to greater limitation to exercise
Clavario et al. (154) 3 months (*n* = 200)	_PEAK_V′O_2_ < or >85% pred	● 49.5% of patients (_PEAK_V′O_2_ <85% pred) ● 61% normal lactate threshold, among those: ◊ 14.8% respiratory limitation ◊ 34.4% cardiac limitation ◊ 50.8% non-cardiopulmonary limitation ● Predictors of low V′O_2_: FEV1, DLCO% pred, and maximal muscle strength ● 80% had one disabling symptoms and was not related to lower _PEAK_V′O_2_
Barbagelata et al. (185) 80 ± 21 days after COVID-19 (*n* = 200)	Post-COVID-19 syndrome (PASC 56%) and asymptomatic post COVID	● _PEAK_V′O_2_ 27.2 ± 8.9 ml/min/kg ● Lower _PEAK_V′O_2_ ● More symptoms during the CPET ● Lower probability of reaching AT ● 89.5% normal O_2_ pulse and 44.5% normal OUES ● Patients with PASC compared to asymptomatic patients had 3.2 ml/min/kg less _PEAK_V′O2 (95% CI −0.9 to −5.5)
Singh et al. (159) 11 months (*n* = 10)	Unexplained exercise intolerance vs. control participants	● Reduced _PEAK_V′O_2_ (70 ± 11% pred) ● Normal oxygen delivery (DO_2_) ● Reduced systemic oxygen extraction (EO_2_) ● Ventilatory inefficiency (high V′E/V′CO_2_ 35 ± 5) with a normal decrease in dead space ventilation
Rinaldo et al. (149) 3 months (*n* = 75)	The severity of hospitalization: mild-moderate, severe, and critical	● 54% of patients (_PEAK_V′O_2_ <85% prev) ● Older and had greater residual pulmonary sequelae ● No difference in lung function ● There was no difference in peakV′O_2_ related to cardiocirculatory and gas exchange responses ● Mild increase of V′E/V′CO_2_ in the critical vs. mild-moderate group
Acar et al. (155) >3 months (*n* = 51)		● _PEAK_V′O_2_ 85 ± 10% pred (or 24 ± 4.6 ml/kg/min) ● No difference in acute disease severity ● Lower slope V′O_2_/WR (5.6 ± 1.4 ml/min/W)
Mancini et al. (186) >3 months after the onset of symptoms (*n* = 71)	Post-COVID-19 vs. control	● Lower _PEAK_V′O_2_ ● Lower anaerobic threshold ● Lower HR and oxygen pulse ● Lower cardiac output during exercise ● Higher peak O_2_ extraction ● No difference in V′E/V′CO_2_, f, and VT
Cassar et al. (156) 2–3 and 6 months (*n* = 58)	Post-COVID and controls	● Reduced _PEAK_V′O_2_ at 2–3 months By 6 months, V′O_2_ improved (31% persisted with lower V′O_2_) but was still reduced relative to controls ● V′E/V′CO_2_ abnormal at 2–3 months and improved at 6 months ● Lower O_2_ pulse at 2–3 months with improvement at 6 months and comparable to controls ● Slower HRR at 2–3 months and comparable to controls at 6 months ● Severity was not associated with lower V′O_2_ ● Improvement of CPET and MRI were not correlated with cardiopulmonary symptoms
Szekely et al. (187) 3–15 months (*n* = 41)	Normal breathing pattern vs. dysfunctional breathing	● 59% of patients (_PEAK_V′O_2_ <80% pred) ● Only 2 CPET (5%) were considered normal ● Dysfunctional breathing (*n* = 26) ● 5 patients with preload failure and symptoms of ME/CFS
de Boer et al. (188) 6 ± 4 months (*n* = 50)	PASC	● 32% of patients (_PEAK_V′O_2_ ≤84% pred) ● None ventilatory limitation ● 56% cardiovascular limitation ● Higher arterial lactate levels and low FATox
Vonbank et al. (157) 3–6 months (*n* = 100)	Moderate/critical vs. asymptomatic/mild vs. normal control	● Lower _PEAK_V′O_2_ and _*atAT*_ ● Lower work peak ● Lower lactate level ● Lower HR ● Higher V′E/V′CO_2_
Ladlow et al. (189) >6 months (*n* = 205)	25% dysautonomia vs. non-dysautonomia	● HR _atrest_ and HR _atAT_ were higher ● Slowly HRR ● Lower work rate _at AT_ and _PEAK_ with same lactate level ● Lower V′O_2 at AT_ and _*at peak*_ exercise ● Mild elevated V′E/V′CO_2_
Ambrosino et al. (190) >2 months (*n* = 36)	Normal vs. reduced exercise capacity	● 77.8% of patients (_PEAK_V′O_2_ <20 ml/kg/min) ● Lower lactate threshold ● Lower peak ventilation ● Higher V′E/V′CO_2_ and no VD/VT reduction in 87.5% of patients ● Lower vascular reactivity (FMD) with slope V′E/V′CO_2_ ≥36
Freìsard et al. (191) >6 weeks of persistent dyspnea (median 119 ± 89 days) (*n* = 51)		● Preserved _PEAK_V′O_2_ 22.9 (20.0–25.5) ml/kg/min in DB patients ● DB (chaotic ventilatory pattern) mostly without hyperventilation in 29.4% (*n* = 15) ● Respiratory limitation with gas exchange abnormalities in 54.9% (*n* = 28) ● Normal CPET or O_2_ delivery/utilization impairment 15.7% (*n* = 8) ● _PEAK_V′O_2_ >84% pred and workload at the peak were within the normal range for DB patients. No relation to hyperventilation was found (normal V′E/V′CO_2_ and PaCO_2_ at rest).
Ribeiro Baptista et al. (158) 3 months (*n* = 105)	_PEAK_V′O_2_ < (reduced) or >(normal exercise capacity) 80% pred	● 35% of patients (_PEAK_V′O_2_ <80% pred) ● No difference in acute disease severity ● 38.9% sarcopenia ● Lower lactate threshold and O_2_ pulse (no ventilatory or gas exchange analysis) ● Obs: relation with low V′O_2_ were: reduced pulmonary function (DLCO) and a decrease in muscular mass index
Schaeffer et al. (161) >3 months (*n* = 49)	Fatigue (*n* = 34) or non-fatigue (*n* = 15)	● Lower _PEAK_V′O_2_ in ml/kg/min (not in % pred). Obs: greater proportion of obese patients. ● METs were lower ● Dyspnea intensity ratings, dyspnea intensity-ventilation, and -V′O_2_ slopes were higher ● No difference in mechanical constraint

mMRC, Medical Research Council modified dyspnea scale; peakV′O_2_, peak exercise oxygen consumption; CPET, cardiopulmonary exercise testing; WR, work rate; V′E/V′CO_2_, minute ventilation by carbon dioxide output; DLCO, carbon monoxide diffusion; FEV1, forced expiratory volume in one second; CT chest, chest computed tomography; ICU, intensive care unit; ME/CFS, myalgic encephalomyelitis/chronic fatigue syndrome; FMD, endothelial-dependent flow mediate dilation; MRI, magnetic resonance imaging; PASC, post-acute sequelae of SARS-CoV-2 infection; FATox, beta-oxidation of mitochondrial substrates fatty acids; iCPET, invasive cardiopulmonary exercise testing; AT, anaerobic threshold; DO_2_, oxygen delivery; EO_2_, systemic oxygen extraction; OUES, oxygen uptake efficiency; f, respiratory rate; VT, tidal volume; METS, peak metabolic equivalents of task.

Ventilatory inefficiencies such as an increase in the minute ventilation to carbon dioxide output ratio during exercise or an increase in the dead space to tidal volume observed in the studies may explain the persistent dyspneic symptoms in the long COVID patients (151, 152, 159, 162). These findings can result from increased central chemosensitivity, dysfunctional breathing, and persistent pulmonary or microvascular injury.

### Pulmonary rehabilitation in post-COVID syndrome

After hospital discharge, patients need to follow-up with a multidisciplinary approach to control and improve their symptoms and sequelae. For this reason, referring the patient with persistent symptoms after COVID-19 infection to a pulmonary rehabilitation program is crucial to accelerate the improvement in symptoms and health status and allow the patient to return to a productive life. The main goal of the pulmonary rehabilitation program is to restore physical, psychological, and social functions, improve the quality of life in COVID-19 survivors and decrease the incidence of long-term disabilities (163–165). In a large longitudinal cohort in China, within 1 year after acute infection, most hospital survivors with COVID-19 had an excellent physical and functional recovery over the months and had returned to their original work and life (166). Therefore, follow-up evaluations are needed, and rehabilitation might be helpful in post-COVID-19 patients.

Each patient will need a specific program based on education, self-management, and exercise training. However, data on the efficacy of particular rehabilitation approaches in the acute and post-acute phases are still scarce (167–174). An open randomized clinical trial comprising 72 elderly patients more than 6 months after contracting COVID-19 showed an improvement in pulmonary function, exercise capacity, and quality of life with 6-week pulmonary rehabilitation therapy (174). Other cohort studies yielded similar results (175, 176).

In terms of rehabilitation modality, a randomized controlled trial showed that low-intensity aerobic training combined with resistance training has better effects on handgrip strength, kinesiophobia status, and quality of life than high-intensity aerobic training combined with resistance training in post-COVID-19 older adults with sarcopenia. However, the intragroup analysis showed that both groups had significant improvement in the muscle bulk irrespective of exercise intensity (172). With the relocation of health professionals from outpatient activities to hospitals (176, 177), telerehabilitation was used to provide a much-needed resource to address the needs of COVID-19 survivors. In a prospectively randomized program, patients allocated to virtual and home physical therapy had improved outcomes (168). A more robust study was performed to compare supervised home telerehabilitation program with no rehabilitation for post-COVID-19. In this trial, the tele-rehabilitation program was superior to no rehabilitation in terms of functional exercise capacity by six-minute walk test, dyspnea-free symptoms, and physical quality of life. (170).

Before starting rehabilitation therapy, it is essential to evaluate the exercise capacity, which could be done by field tests, such as the 6-min walking test, shuttle walking test, step test, etc., or a more complex test, such as cardiopulmonary exercise testing. Patients should be reevaluated in 6–12 weeks. Although the exercise program is essential, which includes aerobic and strengthening exercises for peripheral and respiratory muscles, it is vital to include breathing retraining, airway clearance, energy conservation techniques, and psychological counseling (163, 165, 178–181). Referring the patient with persistent symptoms after COVID-19 infection to a rehabilitation program is crucial to accelerate the improvement in symptoms and health status and allow the patient to return to a productive life.

## Conclusion

COVID-19 survivors may present signs of cardiopulmonary sequelae such as persistent lung fibrosis, chronic thrombosis, right ventricular dysfunction, pulmonary hypertension, and exercise intolerance. Pulmonary fibrosis is among the most feared chronic pulmonary complications of COVID-19. A diagnosis of PCILD should be based on clinical, radiological, and pathological findings. The role of treatment with antifibrotic and anti-inflammatory drugs in improving PCILD symptoms remains inconclusive. A substantial proportion of patients with COVID-19 have coagulation abnormalities. Acute pulmonary embolism in COVID-19 can lead to the development of CTED and CTEPH or even other forms of pulmonary hypertension, which could increase in prevalence over time during the COVID-19 pandemic. Right ventricular dysfunction is prevalent in patients hospitalized with SARS-CoV-2 and is more likely related to systemic consequences rather than direct viral myocardial infection. In terms of mechanisms of exercise intolerance after COVID-19 infection, exercise limitation can be due to a central or peripheral cardiocirculatory origin, decreased oxygen uptake, and with or without ventilatory or gas exchange limitation. Referring the patient with persistent symptoms after COVID-19 infection to a pulmonary rehabilitation program is crucial to accelerate the improvement in symptoms and health status and allow the patient to return to a productive life. We anticipate that as the pool of patients recovering from COVID-19 continues to increase, healthcare providers will need to learn to recognize these long-term cardiopulmonary sequelae early and create a management plan to prevent further deterioration and improve the quality of life of these patients. Furthermore, carefully designed research programs and long-term monitoring of these patients will help clinicians to manage these patients in the long run.

## Author contributions

All authors made substantial contributions to the conception or design of the work; or the acquisition, analysis, or interpretation of data for the work, drafted the work or revising it critically for important intellectual content, provided approval for publication of the content, and agreed to be accountable for all aspects of the work in ensuring that questions related to the accuracy or integrity of any part of the work were appropriately investigated and resolved.
